# Studies on Synthetic and Natural Melanin and Its Affinity for Fe(III) Ion

**DOI:** 10.1155/2012/712840

**Published:** 2012-12-06

**Authors:** T. G. Costa, R. Younger, C. Poe, P. J. Farmer, B. Szpoganicz

**Affiliations:** ^1^Department of Chemistry, Federal University of Santa Catarina, CP476, 88040-900 Florianópolis, SC, Brazil; ^2^Department of Chemistry, University of California, Irvine, Irvine, CA 96729, USA; ^3^Department of Chemistry and Biochemistry, Baylor University, Waco, TX 76706, USA

## Abstract

In this work, we measured the metal-binding sites of natural and synthetic dihydroxyindole (DHI) melanins and their respective interactions with Fe(III) ions. Besides the two acid groups detected for the DHI system: catechol (Cat) and quinone-imine (QI), acetate groups were detected in the natural oligomer by potentiometric titrations. At acidic pH values, Fe(III) complexation with synthetic melanin was detected in an Fe(OH)(CatH_2_Cat) interaction. With an increase of pH, three new interactions occurred: dihydroxide diprotonated catechol, Fe(OH)_2_(CatH_2_Cat)^−^, dihydroxide monoprotonated catechol, [Fe(OH)_2_(CatHCat)]^2−^, and an interaction resulting from the association of one quinone-imine and a catechol group, [Fe(OH)_2_(Qi^−^)(CatHCat)]^3−^. In the natural melanin system, we detected the same interactions involving catechol and quinone-imine groups but also the metal interacts with acetate group at pH values lower than 4.0. Furthermore, interactions in the synthetic system were also characterized by infrared spectroscopy by using the characteristic vibrations of catechol and quinone-imine groups. Finally, scanning electronic microscopy (SEM) and energy-dispersive X-ray (EDS) analysis were used to examine the differences in morphology of these two systems in the absence and presence of Fe(III) ions. The mole ratio of metal and donor atoms was obtained by the EDS analysis.

## 1. Introduction

**Figure 1:**
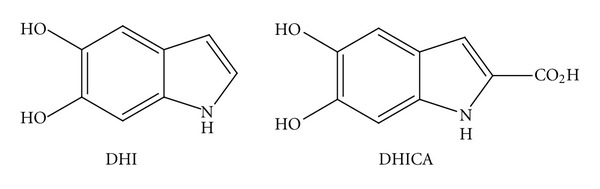
Precursors of the melanin oligomers.

**Figure 2:**
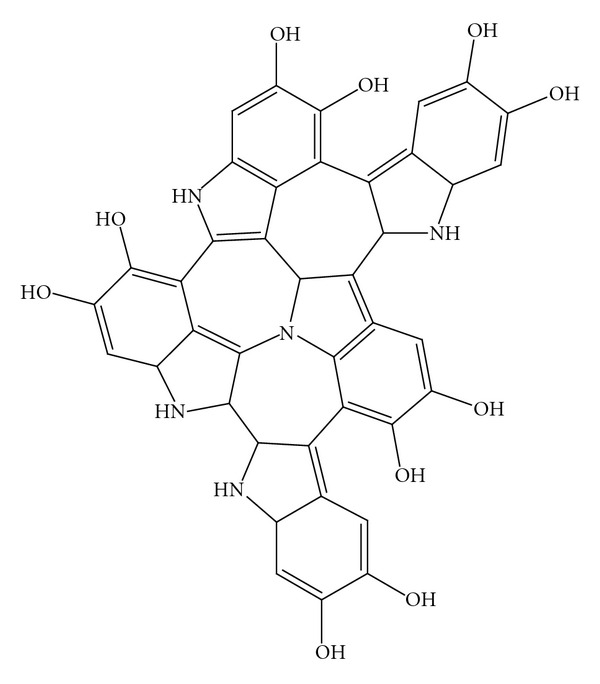
Model of DHI melanin oligomerization.

Melanin is an ubiquitous oligomeric pigment found in plants and in the hair, skin (*eumelanins*), and brain (*neuromelanins*) of animals. The two major chemical monomers are 5,6-dihydroxyindole (DHI) and 5,6-dihydroxyindole-2-carboxylic acid (DHICA) as shown in Figure 1 [1, 2]. The pigment can be synthesized, usually by auto-oxidation of catechols or by tyrosinase-catalyzed oxidation of tyrosine or Dopa [3, 4], to give oligomeric sheets as illustrated in Figure 2. In this work, we also use an enzymatic process for the isolation of *eumelanin* from human black hair, first described by Novellino et al. [5], which allows isolation of intact pigment particles for structural characterization. 

Previous reports have shown that the affinity of divalent metals like Cu(II) and Zn(II) for melanin has a significant effect on the structure of melanin aggregates [6]. The binding of metal ions to melanin also accelerates its bleaching under air or peroxide; for example, melanin-copper complexation has been implicated in Fenton-like catalytic oxidations [7]. Other researchers have studied the binding of Fe(III) by melanins [8–10] and shown that this ion accelerates the air oxidation of DHI and DHICA and suggested that a similar increase in iron-promoted oxidative stress in the substantia nigra is linked to the loss of melanin observed during Parkinson's disease [11–15]. In related studies, we have shown that metal ion complexes, which induce passive uptake of the metal ions into cells, show significant toxicity towards melanin-producing melanoma cells [16].

In this work, we measure the interaction of synthetic melanin (DHI) and natural melanin with Fe(III) ions using potentiometric titrations in combination with mathematical fitting functions. The results are correlated with infrared spectroscopy and scanning electronic microscopy with energy-dispersive X-ray analysis to quantify the binding sites and to see the morphological differences in these types of melanin.

## 2. Experimental

### 2.1. DHI Melanin

 Stock solutions of synthetic melanin were derived from air oxidation of 5,6-dihydroxyindole (DHI), generated in situ by hydrolysis of 5,6-diacetoxyindole (DAI) obtained from TCI America, Portland. The DAI starting material (120 mg, 0.5145 mmol) was treated with 20% molar excess of KOH (1.28 mmol in 250 mL in deionized water), and the mixture was vigorously stirred until the DAI was completely reacted [6]. DAI is not very soluble in water, but as the reaction progresses over 6 hours, all of the DAI is consumed. 

### 2.2. Natural Melanin Extraction

 Hair melanin was obtained by the method described by Novellino et al. [5]. Locks of black hair were washed several times with acetone, distilled water, and a few portions of chloroform. Afterwards, the hair was dried at room temperature and cut to bands of about 5 cm. About 5 g of hair was homogenized with a glass pestle in 50 mL of 0.1 M phosphate buffer at pH 7.5, and the homogenate was submitted to the following treatments: dithiothreitol (0.5 g) was added and the resulting mixture stirred at 37°C under a stream of argon for 18 h. Then, proteinase K (10 mg) and dithiothreitol (0.5 g) were added to the mixture and left stirring at 37°C under argon for an additional 18 h. The mixture was centrifuged for 10 min, and the resulting pellet extensively rinsed with water. The precipitate was then suspended in 30 mL of 0.1 M phosphate buffer pH 7.5, with papain (10 mg) and dithiothreitol (50 mg). This mixture was stirred for 18 h at 37°C under argon and centrifuged as earlier. The black pellet collected, after 6 washings with water, was resuspended in 10 mL of 0.1 M phosphate buffer, pH 7.5, with protease (10 mg) and dithiothreitol (20 mg) added, and the mixture was stirred for 18 h at 37°C under an argon stream. An oxygen-free solution of 2% w/v Triton X-100 was added, and the mixture was stirred for 4 h at room temperature under argon and then centrifuged (4000 rpm) for 20 min. After washing once with water : methanol 1 : 1 v/v, and four times with water, the black pellet was treated again with protease and dithiothreitol as described earlier. The pigment pellet, collected by centrifugation (4000 rpm for 20 min), was dried over NaOH in presence of CaCl_2_ for 24 h to give 200 mg of melanin.

### 2.3. Potentiometric Titration

 The potentiometric studies were carried out in water solution with a Metrohm Titrino plus 350 automatic titrator combined with an Ag/AgCl electrode. The experimental samples were analyzed in a 50 mL sealed thermostated cell that was maintained at 25°C, bubbled with argon to ensure an inert atmosphere. The pH of the experimental solution (20 mL of DHI melanin) was adjusted to 11 with 0.100 M KOH (CO_2_ free, Backer Dilut-It) and back-titrated with 0.100 M HCl. For the native melanin, a 40 mg sample of the melanin particles extracted from hair was dispersed in 20 mL of twice-distilled water. For the Fe(III)-treated samples, melanin solutions were titrated in the presence of metal ions in melanin : metal ion molar ratio of 2 : 1. The Fe(III) solutions were standardized with EDTA [17], and the amount of added HCl to avoid metal hydrolysis was determined by Gran's Plot [18]. The melanin samples were homogenized for 30 minutes to guarantee total equilibration before measurements. The pH of the experimental solutions was brought to 11.0 and back titrated with aliquots of 0.100 M HCl until pH 3.0. The time to reach the equilibrium in each experimental data was about 15 minutes. 

### 2.4. Infrared Studies

 For the IR studies, synthetic melanin was first complexed with Fe(III) by Korytowski method [19] with little modification; this method is used for complexes formed with oligomers and bimolecular systems. A portion of stock DHI solution (40 mL) was mixed with 5 mL of 0.01 M FeCl_3_ solution, and the pH of solutions was brought to 4.0 and 10.0, adjusted by 0.1 M HCl or 0.1 M NaOH solution. FeCl_3_ *·*6H_2_O, (Vetec Ltd.) was used. The mixtures after 3 h were precipitate at 0°C and filtered, the solid was washed with twice-distilled water and dried at vacuum.

The analysis was carried out in KBr pellets with approximately 5–10 mg of the samples at spectrometer Perkin-Elmer FT-IR 1600 with computer detect system, in the region of 500 to 4000 cm^−1^. 

### 2.5. Scanning Electron Microscopy and EDS Analysis

 The samples with pure synthetic melanin and those coordinated to Fe(III) (as described in 2.4) were prepared with silver glass and coated with gold. A Philips XL 30 SEM was used to examine the samples at 30 kV and a 20° tilt. Images were captured using the digital software image acquisition, accolade with the SEM. The EDS spectrum was obtained in the same instrument.

### 2.6. Electrochemical Studies

A 6.0 mg portion of 5,6-diacetoxyindole was dissolved in 12.5 mL degassed ethanol in a glovebox under nitrogen, to which 0.55 mL 0.1 M NaOH was added. The solution was stirred and stood for 1 hour, then diluted up to 25 mL with pH 7.0, 50 mM phosphate buffer. An ITO plate was submerged in the solution, and polymerization of DHI on the plate was done using cyclic voltammetry for 15 cycles at a sweep rate of 50 mV s^−1^ from −0.4 V to 0.6 V versus Ag/AgCl. Solutions of 0.1 M CuCl_2_, ZnSO_4_·7H_2_O, FeCl_3_, and Fe(SO_4_)_2_(NH_4_)_2_·6H_2_O were prepared in atmosphere, degassed, and loaded into glovebox. Freshly made poly-DHI films were soaked in the metal ion solutions overnight, then rinsed with deionized water and stored under nitrogen. UV-Vis spectra were recorded using a 2-neck UV-Vis electrochemical cell in a solution of 0.1 M LiCl, pH 7, 50 mM phosphate buffer. Electrodes were attached, and spectroelectrochemical measurements were made using bulk electrolysis at various potentials versus Ag/AgCl.

## 3. Results and Discussion

### 3.1. DHI Melanin System

**Figure 3:**
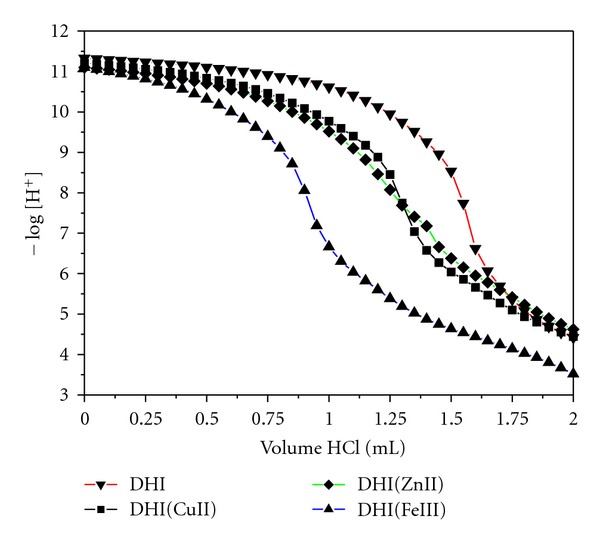
The titration curves of DHI melanin in the absence and presence of metals.

The titration curves for the DHI melanin in the presence and absence of Cu(II), Zn(II), and Fe(III) are shown in Figure 3. The buffer region above pH 9 in the free DHI melanin can be attributed to the acid-base equilibrium involving catecholic groups which are present in melanin. The strong buffer below pH 7 can be attributed to the acid-base equilibrium of quinone-imine group [6]. The acetate molecules present in the solution due to DAI hydrolysis were included in the calculation of the DHI pKa's and the interaction constants with Fe(III) ion. The amount of each group was calculated using the best-fit equilibrium constants in the Best7 program. Also the interaction distribution curves of the Fe(III)-complexed sites in the DHI-melanin were calculated using the Species program [20]. 

**Scheme 1:**
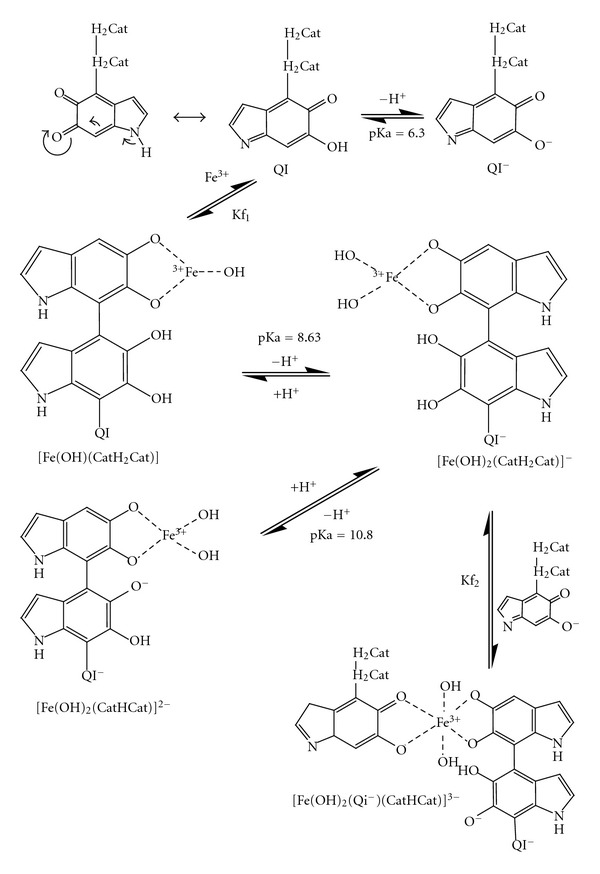
Chemical equilibriums involving all of the species detected, where QI/QI- and H_2_Cat/HCat^−^/Cat^2−^ are protonated and deprotonated forms of quinone-imine and catechol groups.

The metal curves (Figure 3) are below the free DHI melanin curve. The curve in the presence of Fe(III) is much lower than those in the presence of Cu(II) and Zn(II) ions, indicating that the Fe(III) ion interacts more strongly than Cu(II) or Zn(II) ions with melanin. The experimental data was analyzed with the BEST7 [20] program and the equilibrium constants that were determined are shown in Table 1. The equilibrium constants of the interactions with Cu(II) and Zn(II) ions were calculated previously [6]. The equilibriums detected in the DHI system with Fe(III) are shown in Scheme 1.

**Figure 4:**
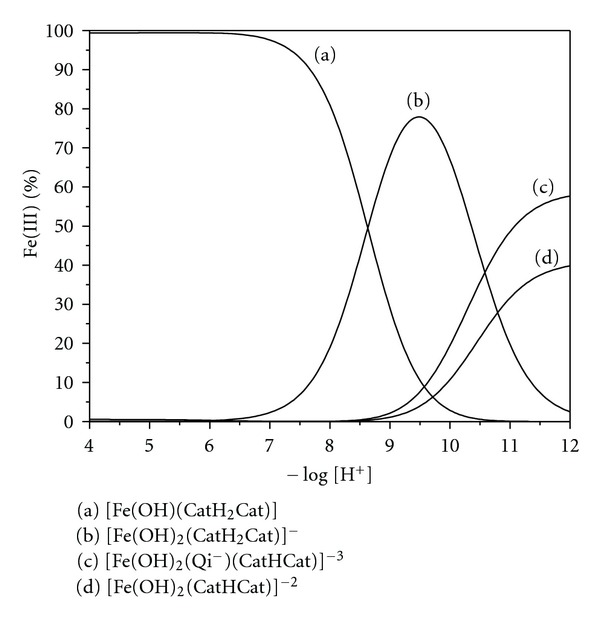
Interaction distribution curves of a DHI melanin solution containing 2.00 mM of DHI melanin (in a 2.0 : 0.5 ratio of catechol:quinone-imine) and 0.99 mM of Fe(III) ions at 25.0°C.

The distribution curves of the interactions in the DHI melanin system (Figure 4) show that at acidic pH values, the monohydroxide Fe(OH)(CatH_2_Cat) interaction predominates. At higher pH values, the dihydroxide [Fe(OH)_2_(CatH_2_Cat)]^−^ interaction increases and is the predominant interaction at pH 8.4–10.3. At pH values above 9, two other interactions appear: the dihydroxide Fe(III) monoprotonated catechol, [Fe(OH)_2_(CatHCat)]^−^, and the dihydroxide Fe(III) quinone-imine monoprotonated catechol, [Fe(OH)_2_(Qi^−^)(CatHCat)]^3−^. These equilibriums are shown in Figure 4.

### 3.2. Natural Melanin System

**Figure 5:**
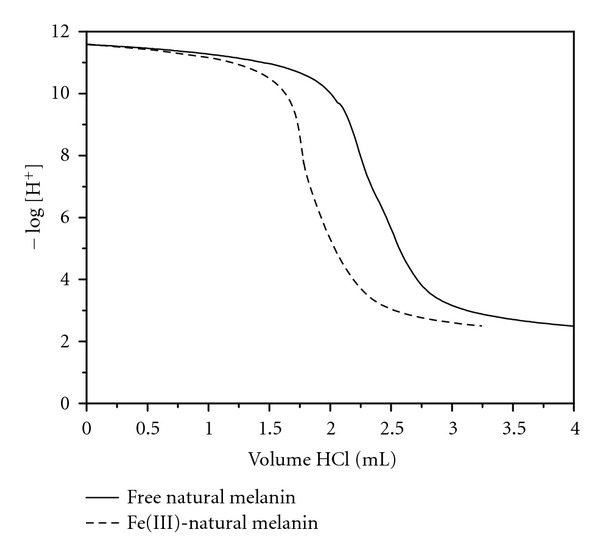
The titration curves for natural melanin in the absence and presence of Fe(III).

As with synthetic melanin, the natural melanin shows the same buffer region above pH 9 which is a characteristic of the presence of catechol groups (Figure 5). The interaction of Fe(III) ion is shown by comparing the two curves (in the presence and absence of the metal ion). Also, we can see the buffer in pH 6 on the melanin Fe(III) curve; it represents the complexation of Fe(III) with the quinone-imine groups. 

In natural melanin, the major group is catechol, and the amount of this group found by potentiometric titration is 3.977 mmol per gram of melanin; the explanation for the major group is that the precursor of natural melanin is the natural amino acid tyrosine, which by hydroxylation reaction catalyzed by tyrosinase leads to the formation of catechol groups in the melanin structure. We also detect quinone-imine 0.300 mmol per gram and 1.188 mmol of acetate groups per gram of melanin. The pKa values for the major groups present in the natural melanin are given in Table 2. 

**Figure 6:**
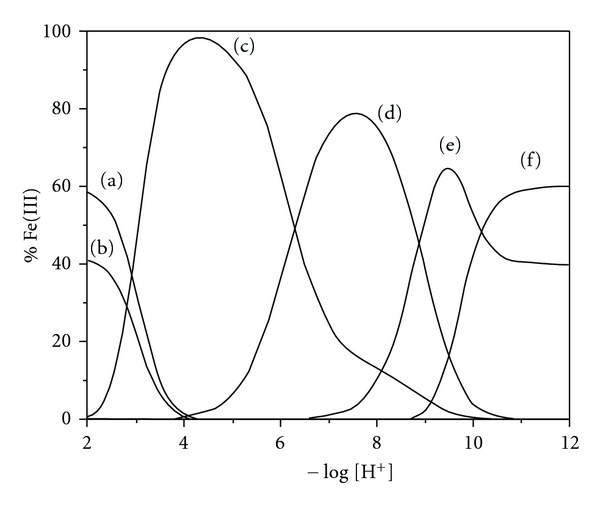
Interaction distribution curves of a natural melanin solution containing 0.00994 mmol of Fe(III) ions and 0.0370 mmol of melanin (40 mg of natural melanin) at 25.0°C., where (a) = Fe(Ac)^2+^, (b) = Fe(Qi)^2+^, (c) = Fe(OH)(Cat), (d) = Fe(OH)(Cat)(Qi)^−^, (e) = Fe(OH)_2_(Cat)(Qi)^2−^, and (f) = Fe(OH)_2_(Cat)_2_^3−^.

The distribution curves of the interactions detected by potentiometric titration are shown in Figure 6. The interactions detected in low pH values 2–4 are resulting from the competition of Fe(III) by quinone-imine, acetate, and catechol groups (a, b, and c), represented by Fe(Ac)^2+^, Fe(Qi)^2+^, and Fe(OH)(Cat)^+^. The interactions of Fe(III) with catechol group predominate at pH values above 3.0. Those interactions are represented by Fe(OH)(Cat), Fe(OH)(Cat)(Qi)^−^, Fe(OH)_2_(Cat)(Qi)^2−^, and Fe(OH)_2_(Cat)_2_^3−^, and the equilibrium constants of all interactions detected are shown in Table 3.

The strong complexation of melanin by Fe(III) in solution was shown by Franz and colleagues [8] using brain melanins, *neuromelanins*, where it was found that acidic pH in the melanin is 1 : 1 coordination with Fe(III). In our work, we detail the coordinate groups in this pH range in this proportion: Fe(Ac)^2+^, Fe(Qi)^2+^, and Fe(OH)(Cat)^+^, at pH 4–8, the formation of melanin 2 : 1 species melanin : metal, in our system represented by the species Fe(OH)(Cat)(Qi)^−^, and higher pH values promote the formation of dihydroxy species.

### 3.3. Infrared Studies

**Figure 7:**
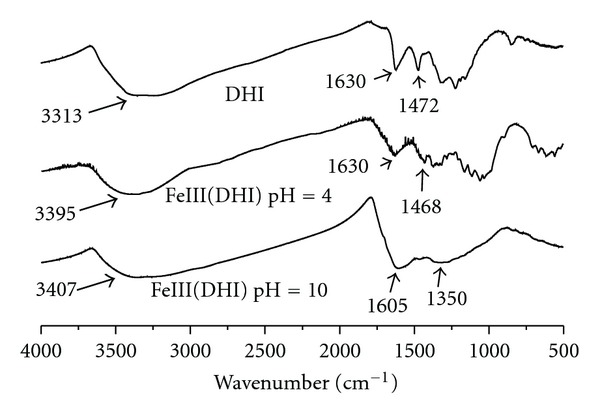
IR spectra of synthetic melanin in the absence and presence of Fe(III) ions at different pH values.

 The IR spectra of synthetic melanin in the absence and presence of Fe(III) ions are shown in Figure 7. Shurygina et al. [21] did one of the first IR analyses of melanin and identified three characteristic peaks: ~3300 cm^−1^, representative of catechol groups, ~1625 cm^−1^, representative of aromatic C=C and/or carboxylate groups as well as nitrogen containing heterocycles, and ~1470 cm^−1^, representative of o-hydroxy quinone groups. The complexation of Fe(III) is identified by the shift of the 3313 cm^−1^ peak, characteristic of melanin in the absence of Fe(III), to 3395 cm^−1^ at pH 4 and 3407 cm^−1^ at pH 10 for melanin in the presence of the metal ion. The presence of quinone-imine is shown at 1472 cm^−1^ for melanin alone. In the presence of metal ions, this peak shifts to lower wavenumbers (1468 cm^−1^ at pH 4 and 1350 cm^−1^ at pH 10). A shift in the C=O stretching in organic compounds to lower wavenumbers by metal complexation was reported by Nakamoto et al. [22]. The interaction of Fe(III) with the quinone-imime group was found in the potentiometric study (Figure 3). 

### 3.4. Scanning Electron Microscopy and EDS Analysis

**Figure 8:**
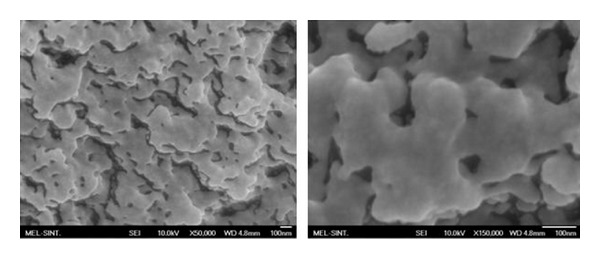
SEM images of synthetic melanin.

**Figure 9:**
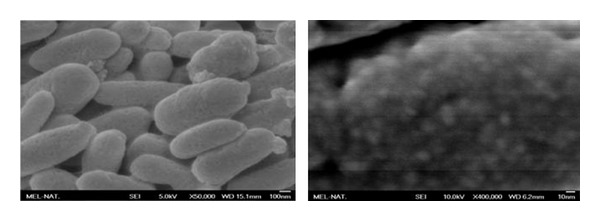
SEM images of the natural melanin used in the experiments.

**Figure 10:**
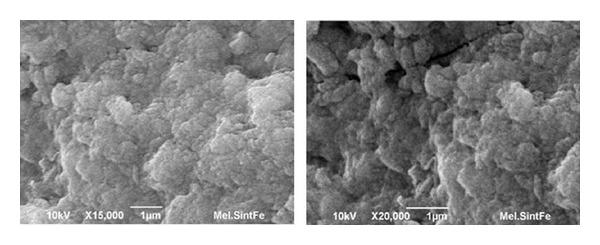
SEM images of the synthetic melanin complex with Fe(III).

The scanning electron microscopy images of synthetic and natural melanin are shown in Figures 8 and 9. Simon et al. [23] used SEM to examine the structure of natural and synthetic eumelanins. It was reported that the synthetic samples appear to be amorphous solids while the natural samples appear to be small spheres. In our studies, the synthetic melanin appears to be amorphous, organized layers of melanin. The natural melanin extracted from hair appears to be small batons rather than spheres as reported in the literature [18]. In the presence of the Fe(III) ions, the layers of synthetic melanin are destroyed (Figure 10), and the amorphous organization predominated.

### 3.5. EDS Analysis

#### 3.5.1. Melanin Complexes with Fe(III)

**Figure 11:**
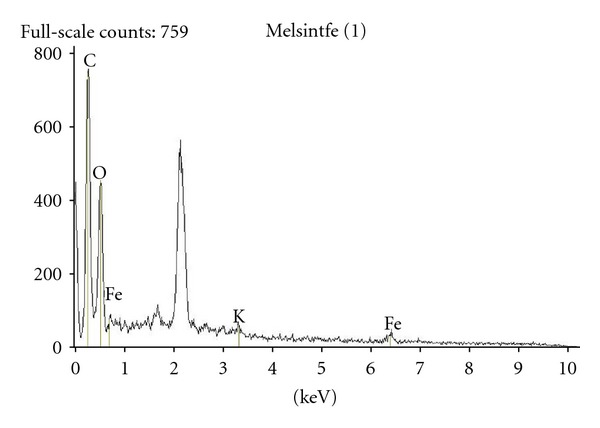
EDS analysis of Fe(III)-DHI.

 Potentiometric results at pH 7 for the catechol complex, [Fe(OH)(CatH_2_Cat)], with a C :  Fe atom ratio of 15 : 1 are shown in Scheme 1.  Table 4 shows semiqualitative analysis, and Figure 11 shows the EDS spectra of the DHI melanin complexed with Fe(III), which gives a 15.1 : 1 atom ratio for C : Fe (atom % column), confirming the potentiometric results. These results confirm that the structure remains the same in both solution and solid states. The presence of potassium is due to isolation of the solid in presence of KCl, and the peak at 2 keV is due to Au, that was used as sample support for EDS analysis.

### 3.6. Electrochemical Studies

The redox activity of melanin is difficult to study in solution, [6, 7] but it has been shown that melanin can be polymerized onto an electrode, whereby the electrochemical properties of melanin can be better studied [7, 24]. Indium-tin-oxide (ITO) electrodes coated with a polymerized DHI film and treated with Cu(II) and Zn(II) had been previously studied [7]. For comparison, treatments of synthetic melanin with Cu(II), and with Fe(II) and Fe(III) as well, were characterized both by UV-Vis spectroscopy and spectroelectrochemical equilibrium studies.

**Figure 12:**
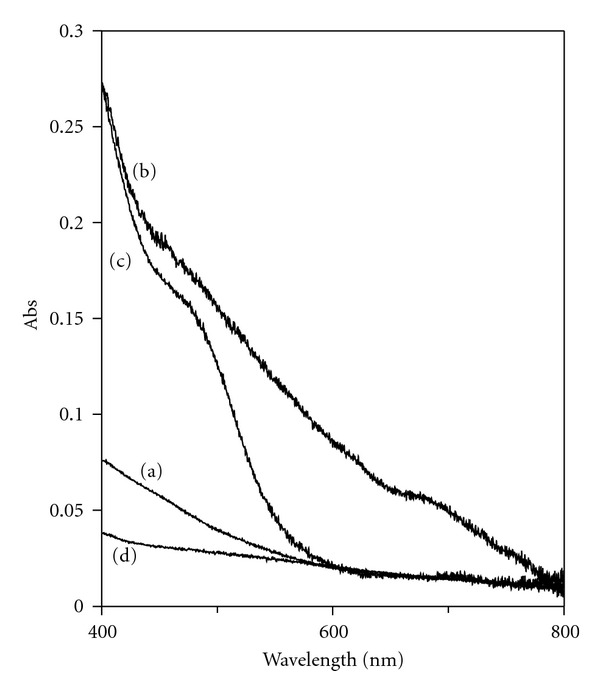
UV-Vis spectra of (a) poly-DHI film, (b) Cu-treated poly-DHI film, (c) Fe(III)-treated poly-DHI film, and (d) Fe(II)-treated poly-DHI film.

Simply treating the poly-DHI ITO plates with metal ion solution tended to increase the overall absorbance of the melanin, as shown in Figure 12. The Fe(III)-treated plates had an absorbance at ~500 nm while the Cu(II)-treated plates shad an additional absorbance at ~680 nm. These results are consistent with previous reports, which were interpreted as metal ion coordination changing the equilibration of the QI/Cat redox states within the melanin, thus affecting its strong absorbance at 500 nm [7]. Treatment of the poly-DHI films with Fe(II) caused a loss of film stability; the films degraded and tended to delaminate off the surface even when handled anaerobically. But notably, measurements on Fe(II)-treated films showed a loss of absorbance at 500 nm. 

**Figure 13:**
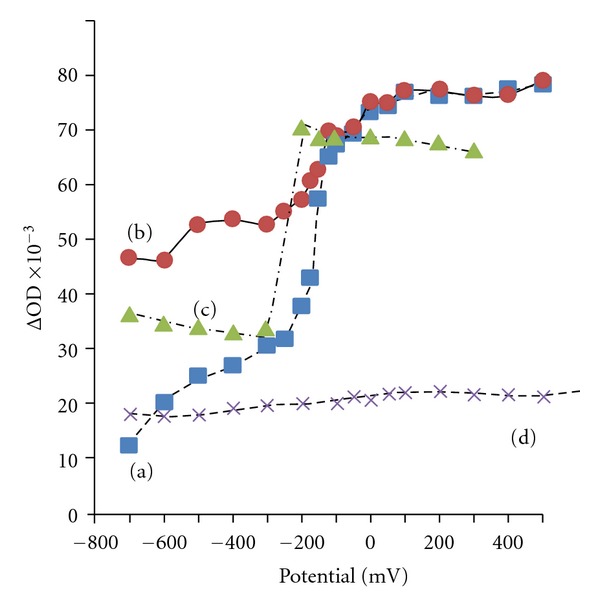
Plot of absorbance at 500 nm versus applied potential for (a) poly-DHI film, (b) Cu-treated poly-DHI film, (c) Fe(III)-treated poly-DHI film, and (d) Fe(II)-treated poly-DHI film.

 The change in absorbance at 500 nm between poly-DHI melanin plates with and without applied potential from −700 to +500 mV is shown in Figure 13. These absorbance values are obtained after the poly-DHI plate electrodes have equilibrated at the applied potential, typically 10 min after initial charging. The resulting spectroelectrochemical absorbance versus potential curves can be interpreted as the effect of metal ion binding on the different redox states of the melanin. The behavior for poly-DHI melanin and the Cu-treated melanin is similar, with two apparent redox equilibria (ca. −150 mV and between −500 and −600 mV). 

The behavior of the Fe(III)-treated sample is unique, and a very distinct transition is observed at about −250 mV, at lower potential than similar transitions for the poly-DHI and Cu-treated melanins. The sharp absorbance increase within about 60 mV span also implies a uniformity in the Fe binding sites within the poly-DHI, which is in agreement with the SEM and EDS results which suggest that the melanin reorganizes upon exposure to Fe(III), perhaps a greater binding affinity for one of quinone tautomers. The lower apparent *E*_1/2_ value for Fe(III)-treated melanin indicates that it is more easily reduced, but again we attribute this to a melanin-based reduction and not one involving the Fe(III/II) redox couple. 

## 4. Conclusion

 In this work, potentiometric titrations, combined with IR spectroscopy and EDS, were used to quantify donor groups and characterize the interactions of synthetic and natural melanin with Fe(III). This ion has a great affinity for the donor groups of both melanins, which is reflected by the equilibrium constants of the interactions. Catechol is the major group interacting with Fe(III) in both forms of melanin. For natural melanin, the interaction products at neutral and basic pH values are [Fe(OH)_2_(Cat^2−^)]^−^ and [Fe(OH)_2_(Cat^−^)_2_]^3−^, respectively. In this system appears the species [Fe(Qi)]^2+^ at pH above 4, but in the DHI melanin, the species present in this pH was not calculated because of the precipitation of the oligomers. The IR spectroscopy results show the characteristic bands for the formation of the species: 3300 cm^−1^ attributed to catechol group, 1625 cm^−1^ and 1470 cm^−1^ attributed to the quinone-imine group, and the interactions of these groups with the metal ions. 

 The SEM images show that synthetic DHI melanin is an amorphous solid while natural melanin has a baton shape. However, in the presence of Fe(III) ion, the melanin layers are destroyed and an amorphous organization is kept. Electrochemical measurements confirm that the melanin is reorganized by treatment with Fe^3+^. EDS analysis shows the mole ratio of atoms present in the metal system, confirming the results of Fe(OH)(CatH_2_Cat) interaction that were detected by potentiometric titrations. 

## Figures and Tables

**Table 1:** Equilibrium constants for the interactions of DHI melanin-Fe(III) systems*.

Equilibria	log⁡*K* (SD)
[Fe(OH)(CatH_2_Cat)][H^+^]/[Fe^3+^][CatH_2_Cat^2−^]	42.52 (1.03)
[Fe(OH)_2_(CatH_2_Cat)]^−^[H^+^]/[Fe(OH)(CatH_2_Cat)]	−8.63 (0.11)
[Fe(OH)_2_(CatHCat)^2−^[H^+^]/[Fe(OH)_2_(CatH_2_Cat)^−^]	−10.80 (0.09)
[Fe(OH)_2_(Qi^−^)(CatHCat)]^3−^/[Fe(OH)_2_(CatH_2_Cat)^2−^][Qi^−^]	5.0 (0.2)

*Obtained by fitting the average of three or more titrations. SD is standard deviation.

**Table 2:** Dissociation constants of major groups in natural melanin*.

Equilibria	−log⁡K_*a*_ (SD)
[Ac^−^][H^+^]/[HAc]	4.35 (0.10)
[Qi^−^][H^+^]/[HQi]	6.30 (0.08)
[HCat^−^][H^+^]/[H_2_Cat]	10.50 (0.12)
[Cat^2−^][H^+^]/[HCat^−^]	12.81 (0.09)

*Obtained by fitting the average of three or more titrations.

**Table 3:** Equilibrium constants for the interactions of natural melanin-Fe(III)*.

Equilibrium	log⁡K (SD)
[Fe(Ac)]^2+^/[Fe^3+^][Ac^−^]	5.20 (0.20)
[Fe(Qi)^2+^]/[Fe^3+^][Qi^−^]	5.73 (0.22)
[Fe(OH)(Cat)][H^+^]/[Fe^3+^][Cat^2−^]	23.53 (0.18)
[Fe(OH)(Cat)(Qi)^−^]/[Fe(OH)(Cat)][Qi^−^]	5.66 (0.15)
[Fe(OH)_2_(Cat)(Qi)^2−^][H^+^]/[Fe(OH)(Cat)(Qi)^−^]	−8.89 (0.05)
[Fe(OH)_2_(Cat)_2_^3−^][H^+^]/[Fe(OH)(Cat)][Cat^2−^]	4.1 (0.08)

*Obtained by fitting the average of three or more titrations.

**Table 4:** EDS results for the Fe(III)-DHI system.

Element line	Weight %	Weight % error	Atom %	Atom % error
C	60.54	±0.98	75.96	±1.23
O	19.70	±0.59	18.55	±0.56
K	1.29	±0.25	0.50	±0.10
Fe	18.48	±2.14	4.99	±0.58

Total	100.00		100.00	
